# Alterations in fatty acid metabolism in response to obesity surgery combined with dietary counseling

**DOI:** 10.1038/nutd.2017.33

**Published:** 2017-09-04

**Authors:** P Walle, M Takkunen, V Männistö, M Vaittinen, P Käkelä, J Ågren, U Schwab, J Lindström, J Tuomilehto, M Uusitupa, J Pihlajamäki

**Affiliations:** 1 Institute of Public Health and Clinical Nutrition, University of Eastern Finland, Kuopio, Finland; 2 Department of Medicine, University of Eastern Finland and Kuopio University Hospital, Kuopio, Finland; 3 Department of Surgery, University of Eastern Finland and Kuopio University Hospital, Kuopio, Finland; 4 Institute of Biomedicine, University of Eastern Finland, Kuopio, Finland; 5 Clinical Nutrition and Obesity Center, Kuopio University Hospital, Kuopio, Finland; 6 National Institute for Health and Welfare, THL, Helsinki, Finland; 7 Center for Vascular Prevention, Danube University Krems, Krems, Austria; 8 Diabetes Research Group, King Abdulaziz University, Jeddah, Saudi Arabia; 9 Dasman Diabetes Institute, Dasman, Kuwait

## Abstract

**Background:**

The effects of obesity surgery on serum and adipose tissue fatty acid (FA) profile and FA metabolism may modify the risk of obesity-related diseases.

**Methods:**

We measured serum (*n*=122) and adipose tissue (*n*=24) FA composition and adipose tissue mRNA expression of genes regulating FA metabolism (*n*=100) in participants of the Kuopio Obesity Surgery Study (KOBS, age 47.2±8.7 years, BMI 44.6±6.0, 40 men, 82 women) before and one year after obesity surgery. As part of the surgery protocol, all the subjects were instructed to add sources of unsaturated fatty acids, such as rapeseed oil and fatty fish, into their diet. The results were compared with changes in serum FA composition in 122 subjects from the Finnish Diabetes Prevention study (DPS) (age 54.3±7.1 years, BMI 32.2±4.6, 28 men, 94 women).

**Results:**

The proportion of saturated FAs decreased and the proportion of n-3 and n-6 FAs increased in serum triglycerides after obesity surgery (all *P*<0.002). Weight loss predicted changes in quantitative amounts of saturated FAs, monounsaturated FAs, n-3 and n-6 FAs in triglycerides (*P*<0.002 for all). Moreover, the changes in adipose tissue FAs reflected the changes in serum FAs, and some of the changes were associated with mRNA expression of elongases and desaturases in adipose tissue (all *P*<0.05). In line with this the estimated activity of elongase (18:1 n-7/16:1 n-7) increased significantly after obesity surgery in all lipid fractions (all *P*<4 × 10^−7^) and the increase in the estimated activity of D5D in triglycerides was associated with higher weight loss (*r*=0.415, *P*<2 × 10^−6^). Changes in serum FA profile were similar after obesity surgery and lifestyle intervention, except for the change in the absolute amounts of n-3 FAs between the two studies (*P*=0.044).

**Conclusions:**

Beneficial changes in serum and adipose tissue FAs after obesity surgery could be associated with changes in endogenous metabolism and diet.

## Introduction

The role of fatty acids (FAs) in obesity-related morbidity is of great interest, and the circulating FA profile is associated with various metabolic disorders such as insulin resistance, type 2 diabetes, low-grade inflammation and obesity itself.^1, 2, 3, 4, 5, 6, 7^ FAs in circulating blood are partly derived from diet,^8^ but the FA composition is also strongly affected by the activities of FA elongases and desaturases that are known to be regulated by insulin.^1, 9^ Therefore, insulin resistance with alterations in endogenous FA metabolism may explain some of the obesity-related changes in FA levels.

Roux-en-Y gastric bypass (RYGB) has a capability to induce sustained weight loss and improve insulin action in body tissues.^10, 11^ RYGB is a combined malabsorptive and restrictive procedure and it has been shown to induce reductions in serum triglycerides, cholesterol and cholesteryl esters.^12, 13, 14^ When compared with conventional treatment of obesity, bariatric surgery leads to greater long-term weight loss, higher remission of metabolic syndrome and type 2 diabetes, and decrease in cardiovascular risk.^10, 15^ It is still unclear to what extent the effects of RYGB are related to changes in endogenous metabolism, weight loss or changes in the diet.

We propose that altered FA metabolism in serum and adipose tissue might contribute to the metabolic benefits occurring after obesity surgery. We investigated changes in serum FAs in triglycerides (TG), cholesteryl esters (CE) and phospholipids (PL), and adipose tissue FA composition in TG, in response to RYGB. We were also able to study changes in the subcutaneous adipose tissue mRNA expression of selected genes participating in FA metabolism. Finally, we compared the effects of obesity surgery with weight reduction induced by lifestyle changes using the data from the Finnish Diabetes Prevention Study (DPS)^16, 17^ as the reference.

## Materials and methods

### Subjects in the KOBS

All patients undergoing obesity surgery in Kuopio University Hospital are recruited into an ongoing study investigating metabolic consequences of obesity surgery.^18, 19^ The present analysis includes data from 122 people (40 men and 82 women, age 47.2±8.7 years, 47 with type 2 diabetes at baseline, Table 1), who were accepted for RYGB and had participated in both baseline and follow-up visits. In a subset of participants, we measured adipose tissue FA composition (*n*=24) and adipose tissue mRNA expression (*n*=100) of genes regulating FA metabolism before and one year after gastric bypass. As part of the surgery protocol, subjects were instructed to follow a preoperative very low-calorie diet (600–800 kcal) for an average of 4 weeks. After operation, the subjects were instructed to consume 3 tea spoons of rapeseed oil and 6 tea spoons of mainly rapeseed oil based spreads daily, for at least 1–2 years after the obesity surgery. Moreover, they were instructed to consume fish 2–3 times a week. The study protocol was approved by the Ethics Committee of the Northern Savo Hospital District. It was performed in accordance with the Helsinki Declaration. Written informed consent was obtained from all the participants.

### Subjects in the Finnish DPS

A total of 522 people at a high risk of type 2 diabetes participated in the randomized, controlled DPS study (NCT00518167, ClinicalTrials.gov).^16, 17^ The goals of the intensified and individualized lifestyle intervention included the following targets: weight loss of at least 5%, moderate daily physical activity (at least 4 h per week), dietary fat intake below 30%, saturated fat intake below 10% of total energy intake, and the intake of dietary fiber at least 15 g/1000 kcal of daily energy intake. The control group received only general information about the benefits of healthy diet and physical activity annually. One year after the baseline measurements subjects participated in the first annual visit and had a blood sample drawn. The data for serum FA composition have been published before.^20^ In the present study we compared the effects of obesity surgery to successful lifestyle change in the DPS. To have a relevant control group with considerable weight loss for the subjects who underwent RYGB, we pooled the intervention and control groups from DPS and selected 122 subjects (28 men, 94 women, age 54.3±7.1 years, mean BMI 32.2±4.6, Table 1) that had lost most weight (kg) during the first year of the active study period. The DPS study has been performed according to the Declaration of Helsinki. All participants have provided a written informed consent. The study design has been approved by the Ethics Committee of the National Public Health Institute of Finland.

### Clinical measurements and laboratory determinations

BMI was calculated as weight (in kilograms) divided by height (in meters) squared. The clinical measurements in KOBS and DPS have previously been reported.^17, 19^

### Assessment of serum and adipose tissue fatty acid composition and estimation of enzyme activities

In KOBS, the fasting serum samples were extracted with chloroform–methanol (2:1) and the different lipid fractions, TG, CE and PL, were separated by solid phase extraction with an aminopropyl column. The adipose tissue samples were pulverized with liquid nitrogen and ~40 mg of adipose tissue was extracted with chloroform–methanol (2:1) and TG and PL fractions were separated as described above. FAs were analyzed according to previously described methods.^21, 22^ In DPS, the total serum FA composition was measured by TETHYS Bioscience Inc. (Emeryville, CA, USA) in 2010 using serum samples according to previously described methods.^20^ The results of FA analysis are expressed as molar percentages (mol/mol of all fatty acids) or as quantitative amounts (mg l^−1^ in KOBS serum and nmol g^−1^ in DPS). The enzyme activities in serum CE, TG and PL were estimated as product-to-precursor ratios of individual FAs as previously described.^19^

### TruSeq targeted RNA expression

Biopsy samples for gene expression analysis were immediately frozen in liquid nitrogen. Custom gene panel (Illumina, San Diego, CA, USA) was used for measuring gene expression levels in human subcutaneous adipose tissue according to instructions provided by the manufacturer using MiSeq system (Illumina), as described previously.^19, 23^

### Statistical analysis

Data are presented as mean±s.d. A logarithmic transformation was performed for skewed variables. To assess differences between baseline and follow-up measurements, the paired samples *t*-test was used. To adjust the results of analyses of 23 individual FA levels in KOBS and DPS for multiple comparisons, Bonferroni correction was used and thus *P*-value <0.002 was considered statistically significant. Linear regression analysis, with weight loss and serum fasting insulin as independent variables, was used to estimate their effect on serum FA composition. For the analyses of subcutaneous adipose tissue mRNA expression in KOBS, the expression levels for each gene per sample in the custom gene panel were normalized based on the total number of aligned reads of the corresponding sample and the results are shown as percentage of total transcript reads. A nominal *P*-value <0.05 was considered statistically significant. Statistical significance for changes in FAs between the two study groups was calculated with independent samples *t*-test. There was homogeneity of variances for all FAs in independent samples *t*-test, as assessed by Levene's test, except for n-3 FAs (*P*=0.0002). The IBM SPSS Statistics for Windows software, Version 21 (IBM Corp., Armonk, NY, USA), was used for statistical analyses.

## Results

### Characteristics

The characteristics of the KOBS participants at baseline and at follow-up visit one year after gastric bypass are presented in Table 1. In line with previous studies, all parameters except total and low-density lipoprotein (LDL) cholesterol concentrations were significantly improved at one year after bariatric surgery.^24^ Mean weight loss was 30.0 kg (−23.2%) during the first year after surgery.

### Changes in serum fatty acid composition

**Figure 1:**
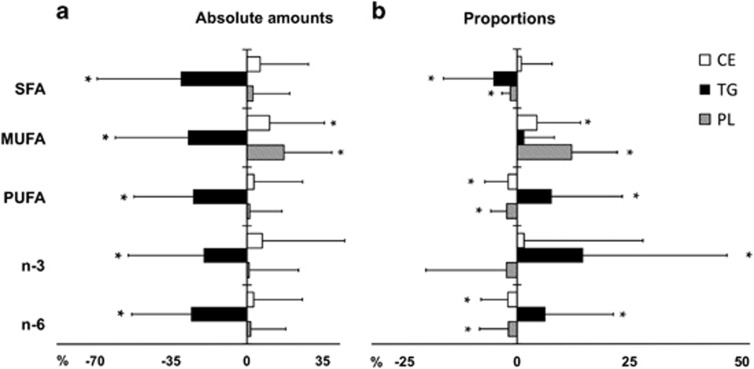
Change in serum fatty acid composition (+s.d.) during the first year after obesity surgery. (**a**) Changes in the absolute amounts (mg l^−1^) of lipid classes and (**b**) changes in fatty acid proportions (mol%). SFA, saturated fatty acids; MUFA, monounsaturated fatty acids; PUFA, polyunsaturated fatty acids; n-3, omega-3 fatty acids; n-6, omega-6 fatty acids. *P<0.002 in paired samples *t*-test.

In TG, the quantitative amounts of saturated FAs (SFAs), monounsaturated FAs (MUFAs) and polyunsaturated FAs (PUFAs) decreased in serum (all *P*<2 × 10^−13^, Figure 1a and Supplementary Table 1). Additionally, the proportion of SFAs decreased (*P*=4 × 10^−8^) and the proportion of n-3 and n-6 FAs in total increased (both *P*<0.0001, Figure 1b, Table 2). In CE and PL, there were no significant changes in quantitative amounts of lipid classes except for the concentration of MUFAs, which increased (both *P*<0.0004, and Supplementary Table 2). However, the proportions of FAs changed markedly both in CE and PL, Figure 1b(Supplementary Table 3). In both fractions, the proportion of MUFAs increased, which was mostly a result of increase in the proportion of oleic acid (18:1 n-9, *P*<0.002). Moreover, the proportions of total PUFAs and n-6 FAs decreased (all *P*<0.001, Supplementary Table 3) in both CE and PL. The proportion of total n-3 FAs did not change in CE or in PL. The data for proportions of individual fatty acids in TG are presented in Table 2 and for the absolute amounts in Supplementary Table 1. Supplementary Table 2 shows the absolute amounts and Supplementary Table 3 the proportions of individual FAs in CE and PL.

### Effect of weight loss and change in fasting insulin concentration on lipid composition

During the first year after obesity surgery subjects lost a significant amount of weight and their fasting insulin concentrations improved (both *P*<5 × 10^−8^, Table 1). A linear regression established that weight loss correlated with changes in quantitative amounts of SFAs, MUFAs, n-3 and n-6 FAs in TG (*P*<0.001 for all) and weight loss accounted for 8–13% of the variability (Supplementary Table 1). In linear regression analysis, the combined effect size of weight loss and change in fasting insulin concentration was only slightly stronger (up to 6%) than weight loss alone (data not shown). Although weight loss predicted the changes in quantitative amounts of lipids, it was not a significant predictor for the relative amounts of serum FAs except for the proportion of SFAs in TG, in which weight loss accounted for 10% of the variability (*P*=2 × 10^−4^, Table 2). Data demonstrating the association between weight loss and serum FAs in CE and PL are shown in Supplementary Tables 2 and 3.

### Changes in subcutaneous adipose tissue FA composition

In addition to serum FAs, we investigated FA composition in TG fraction of the subcutaneous adipose tissue in a subsample of 24 subjects who underwent RYGB (Supplementary Tables 4 and 5). Similarly to serum, the proportion of SFAs in TG was significantly decreased in subcutaneous adipose tissue one year after obesity surgery (*P*=9 × 10^−6^). Although the increase in the proportion of MUFAs in serum TG did not reach significance (*P*=0.019), we observed a significant increase in the proportion of MUFAs in adipose tissue (*P*=2 × 10^−7^).

### Changes in estimated enzyme activities in serum

The estimated activity of elongase (estimated as a ratio of 18:1 n-7/16:1 n-7) increased significantly after obesity surgery in all lipid fractions (all *P*<4 × 10^−7^, Table 2 for TG and Supplementary Table 3 for CE and PL). Weight loss was associated with changes in elongase activity (all *P*<7 × 10^−5^) accounting for up to 27% of the variability (Table 2; Supplementary Table 3). The estimated activity of SCD1 decreased in CE in response to obesity surgery (*P*=3 × 10^−8^, Supplementary Table 3) and decrease in the activity of SCD1 was associated with higher weight loss (*r*=−0.312 in TG, *r*=−0.454 in CE and *r*=−0.369 in PL, all *P*<0.002) The activity of DNL decreased in response to obesity surgery (*P*=7 × 10^−5^, Table 2). There were no significant changes in activities of D5D and D6D in response to obesity surgery, but increase in the estimated activity of D5D was associated with higher weight loss (*r*=0.415 in TG, *r*=0.406 in CE and *r*=0.448 in PL, all *P*<4 × 10^−6^, Supplementary Figure 1). Moreover, higher weight loss associated with lower activity of D6D in CE (*r*= −0.413, *P*=2 × 10^−6^).

### mRNA expression in subcutaneous adipose tissue

We investigated the subcutaneous adipose tissue mRNA expression of genes regulating enzyme activities of elongases and desaturases in response to obesity surgery (Table 3). We discovered that the expression levels of *ELOVL3, ELOVL5* and *ELOVL6* increased similarly to estimated elongase activity (*P*<0.05), while the mRNA expression level of *ELOVL1* decreased during the first year after obesity surgery (*P*=6 × 10^−5^).

### Comparison of obesity surgery and lifestyle intervention

**Figure 2:**
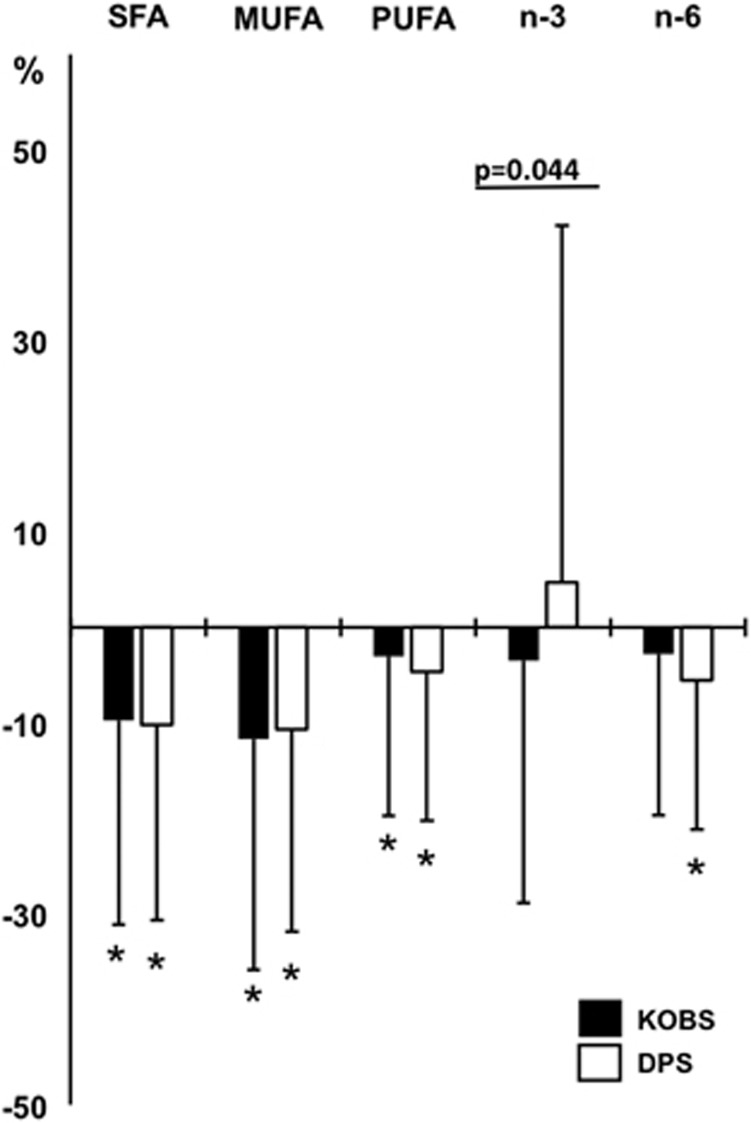
Changes in serum lipid profile after obesity surgery and weight reduction achieved by healthy diet and increased physical activity. Black bars for Kuopio Obesity Surgery Study (KOBS, mean weight loss 23.4%) and white bars for Finnish Diabetes Prevention Study (DPS, mean weight loss 8.4%). SFA, saturated fatty acids; MUFA, monounsaturated fatty acids; PUFA, polyunsaturated fatty acids; n-3, omega-3 fatty acids; n-6, omega-6 fatty acids. Statistical significance between changes in n-3 FAs was calculated with independent samples *t*-test. *P<0.05 in one-sample *t*-test for change in both studies separately.

We compared the effects of obesity surgery with weight reduction achieved by lifestyle intervention in 122 participants from both the KOBS and DPS studies. Mean weight loss in DPS study cohort was 7.4 kg (−8.3%) and concentrations of serum fasting insulin, fasting glucose, total cholesterol, HDL cholesterol and triglycerides improved significantly during the first year of the study (all *P*<0.05, Table 4). As FA composition data were available only for total lipids in the DPS, we had to pool the FAs of TG, CE and PL in the KOBS for comparison (Supplementary Table 6). The main finding was that changes in SFAs, MUFAs and total PUFAs were comparable in response to obesity surgery and lifestyle intervention, with the exception n-3-PUFAs (Figure 2; Supplementary Tables 6 and 7). There was a borderline significant decrease in the amount of n-3 FAs in response to surgery (*P*=0.004, Figure 2). Moreover, there was a significant difference in the changes of n-3 FA levels in response to lifestyle intervention and obesity surgery (*P*=0.044, Figure 2). When the comparison between these two studies was adjusted for weight loss, differences in the changes of SFAs, MUFAs, PUFAs and n-6 FAs between the two studies became statistically significant (Supplementary Table 8). We also discovered that weight loss significantly predicted the changes in the amount of total lipids in all lipid classes after obesity surgery but not after lifestyle change (linear regression analysis, Supplementary Table 9). The changes in estimated enzyme activities in total serum lipids were similar in response to lifestyle change and obesity surgery, as the activity of SCD1 decreased and activity of elongase (18:1 n-7/16:1 n-7) increased (both *P*<0.001, Supplementary Tables 6 and 7). The activity of D5D increased after lifestyle intervention (*P*=0.0002, Supplementary Table 7) but not after obesity surgery. The correlations between weight loss and enzyme activities were similar after lifestyle intervention and obesity surgery, but in DPS the associations did not reach statistical significance (*r*=0.113, *P*=0.216 for D5D and *r*=−0.162, *P*=0.075 for D6D, data not shown).

## Discussion

In this study, we established that obesity surgery combined with dietary counseling leads to potentially beneficial changes in serum FA profile. In addition to weight loss, the observed changes are related to altered endogenous enzyme activities and potential changes in the diet. Our results suggest that changes in fatty acids after obesity surgery were attributed mainly to weight loss, and that these changes, except for the change in n-3 PUFAs, were comparable to changes induced by lifestyle intervention without surgery.

Most of the changes we observed in serum FAs in response to surgery were potentially beneficial, as expected.^12, 14, 25^ Most notably, the proportion of SFAs decreased and PUFAs increased in TGs. There was also an increase in the quantitative and relative amounts of MUFAs in CE and PL, similarly to a previous study.^25^ Obviously, the results of serum CE and PL fraction differed from those of the TG fraction because the total cholesterol concentration was unchanged, whereas concentration of triglycerides decreased significantly.^14, 26^ We suggest that the decrease in serum TGs is primarily related to weight loss, since dietary modifications of fat or carbohydrate intake have often been associated with less significant decreases in serum TGs as observed here in response to surgery.^27, 28, 29^

Interestingly, the change in SFA proportion associated with weight loss, while the changes in the proportions of PUFAs and MUFAs did not, suggesting that dietary intake of PUFAs and MUFAs may be important determinants of serum FAs after surgery.^8, 25^ The proportion of MUFAs was increased mostly due to increase in the level of oleic acid (18:1 n-9), which is one of the major FAs in both CE and PL, and has previously been shown to reflect the change in dietary intake of MUFAs.^8^ Thus, the increase in the proportion of oleic acid (18:1 n-9) could be explained by increased intake of rapeseed oil and vegetable oil based spreads, rich in oleic acid. This is further supported by the fact that the absolute amount and proportion of another major FA in rapeseed oil, α-linolenic acid (18:3 n-3), was increased in both CE and PL. Furthermore, the downstream products of α-linolenic acid, eicosapentaenoic acid (20:5 n-3) and docosapentaenoic acid (22:5 n-3), were increased in TG in response to obesity surgery. Since α-linolenic acid is an essential FA only derived from the diet, the significant increase in its proportion suggests increased dietary intake. Obesity surgery has recently been associated with decreased proportion of eicosapentaenoic acid and unchanged proportion α-linolenic acid, resulting from low intake of n-3 FAs,^25^ but our results suggest that sufficient intake of vegetable oils after obesity surgery increases the proportions of n-3 FAs. On the other hand, the increase in the proportions of eicosapentaenoic acid and docosapentaenoic acid in our study might be related to changes in endogenous metabolism, since high D5D and elongase activities were associated with higher weight loss in our study.

Similarly to serum TG fraction, we observed a significant decrease in the relative amount of SFAs in adipose tissue. The decrease in the proportion of SFAs was mainly due to a decrease in the proportion of palmitic acid, a major FA in adipose tissue. Palmitic acid in adipose tissue is negatively associated with insulin sensitivity,^30^ suggesting that changes induced by obesity surgery and following dietary changes are beneficial. Moreover, both the absolute amount and proportion of oleic acid were increased in adipose tissue TG, as was observed in serum CE and PL. As the FA composition of adipose tissue is considered to reflect the long-term intake of dietary FAs,^8, 31^ and because we observed similar changes in serum and in adipose tissue, these results support our suggestion that the dietary intake of unsaturated FAs was increased as was instructed to study participants.

Changes in serum and adipose tissue FA profile after obesity surgery could also be related to alterations in endogenous metabolism. We observed an increase in the estimated activity of elongase (18:1 n-7/16:1 n-7). This change was strongly associated with weight loss, but might be also related to improved glucose metabolism.^32^ As for desaturases, increased activity of D5D and decreased activities of SCD1 and D6D were linked with higher weight loss after obesity surgery. Weight loss was also associated with a decrease in the estimated activity of serum DNL, indicating reduced endogenous FA synthesis after obesity surgery. However, in addition to weight loss, these decreases SCD1 and DNL activities may also be caused by lower intake of carbohydrates.^33, 34^ Thus, many changes in serum FA profile in response to surgery may be related to changes in endogenous FA metabolism along with changes in the diet, and not to weight loss.

The increase in the expression of several elongase genes in adipose tissue is consistent with earlier reports demonstrating that weight loss, induced by gastric bypass, leads to altered expression and methylation of genes in adipose tissue.^35, 36^ More specifically, we observed increases in the expressions of *ELOVL3, ELOVL5* and *ELOVL6. ELOVL5* plays a role in the elongation of n-3 and n-6 FAs,^37^ and although the proportion of total n-6 FAs was not altered, the proportion of a long-chain adrenic acid (22:4 n-6) was significantly increased in adipose tissue, which could be a result of upregulation of *ELOVL5*. *ELOVL6* elongates palmitic acid to stearic acid which can be desaturated to oleic acid.^37, 38^

Another important observation in our study was that the changes in serum FA profiles were similar after obesity surgery and lifestyle changes leading to 8% weight loss (as compared 23% after gastric bypass), except for n-3 PUFAs. The change in the absolute amounts of n-3 PUFAs differed in response to surgery and lifestyle changes, which may be related to differences in the intake of PUFAs, and to differences in the amount of weight loss, which potentially regulates endogenous enzyme activities, as shown above for mRNA expression of elongases. In fact, weight loss was a significant predictor for FA levels after obesity surgery in all lipid classes but not after lifestyle changes. Although subjects in both studies were instructed to add sources of n-3 FAs into their diets, RYGB has been shown to promote fat malabsorption and thus might result in lower levels of n-3 FAs in patients who have undergone obesity surgery.^25, 39, 40^ Additionally, we observed that the estimated activity of D5D, a key enzyme in the PUFA metabolism,^41^ was increased after lifestyle intervention induced weight loss but not after gastric bypass. However, the amount of lost weight correlated significantly with estimated desaturase activities also after obesity surgery.

We acknowledge the following limitations in our study. The interpretation of our results is complicated by the fact that obesity surgery leads to changes in intestinal anatomy and diet as well as to weight loss. Unfortunately, the methods to assess serum and plasma FAs in the DPS and KOBS studies were slightly different. In the DPS, the FA composition was determined in total lipids instead of separate fractions, and to compare the two studies, we had to pool the FAs of TG, CE and PL in the KOBS. Although this does not provide us with the full data of serum FAs, the combination of these three fractions comprises 94–97% of total plasma fatty acyl chains.^8^ We also acknowledge that the number of subjects with adipose tissue FA data (*n*=24) was low and consequently, some tests lacked statistical power. However, we felt that even though the study population with adipose tissue biopsies was small, it provided valuable information as we could confirm many of the changes in serum FA profile also in adipose tissue.

In conclusion, we observed beneficial changes in FA profile that associate with alterations in endogenous FA metabolism in response to surgery and with changes in the diet. Importantly, we discovered that changes in serum FA profile after obesity surgery are similar to those related to weight reduction achieved by healthy diet and increased physical activity, at least if the intake of vegetable oils and n-3 FAs is sufficient. In future trials, investigating what is sufficient intake of n-3 FAs for patients, who have undergone obesity surgery, will be important.

## Figures and Tables

**Table 1:** Clinical characteristics of the Kuopio Obesity Surgery study (KOBS, *n*=122)

	*Baseline*	*1 year*	P*-value*
Gender (men/women)	40/82		
Age (years)	47.2±8.7		
Weight (kg)	**128.9**±**19.3**	**98.9**±**18.2**	**<0.001**
Body mass index (kg m^−2^)	**44.6**±**6.0**	**34.2**±**5.7**	**<0.001**
Fasting glucose (mmol l^−1^)	**6.4**±**1.5**	**5.5**±**0.9**	**<0.001**
Fasting Insulin (mU l^−1^)	**19.7**±**12.1**	**10.7**±**9.6**	**<0.001**
Total cholesterol (mmol l^−1^)	4.3±1.1	4.4±0.9	>0.05
HDL cholesterol (mmol l^−1^)	**1.1**±**0.3**	**1.5**±**0.4**	**<0.001**
LDL cholesterol (mmol l^−1^)	2.5±0.9	2.4±0.8	>0.05
Triglycerides (mmol l^−1^)	**1.6**±**0.7**	**1.1**±**0.5**	**<0.001**
Alanine aminotransferase (U l^−1^)	**45.7**±**31.0**	**26.7**±**16.1**	**<0.001**

Values are presented as mean±s.d.

Statistically significant changes are in bold (*P*<0.05 in paired samples *t*-test).

**Table 2:** The fatty acid composition (mol%) and estimated enzyme activities in serum triglycerides in the Kuopio Obesity Surgery Study (KOBS, *n*=122)

*Fatty acids and enzyme activities*	*Baseline*	*1 year*	*Paired samples*t*-test*	*Linear regression*		
				*Effect of weight change (%)*		
			P*-value*	*Adjusted*R^*2*^	β	P*-value*
Saturated fatty acids	**32.8**±**3.2**	**30.9**±**3.5**	**<0.002**	**0.10**	**0.33**	**<0.002**
Myristic acid (14:0)	1.6±0.6	1.8±0.9	<0.05	**0.11**	**0.34**	**<0.002**
Palmitic acid (16:0)	**28.1**±**2.4**	**26.1**±**2.5**	**<0.002**	0.06	0.26	<0.01
Stearic acid (18:0)	3.0±0.6	2.9±0.7	<0.05	0.05	0.25	<0.01
						
Monounsaturated fatty acids	49.8±3.0	50.5±3.3	<0.05	0.05	−0.25	<0.01
Palmitoleic acid (16:1 n-7)	**5.2**±**1.3**	**4.7**±**1.4**	**<0.002**	**0.17**	**0.42**	**<0.002**
Vaccenic acid (18:1 n-7)	**3.0**±**0.4**	**2.9**±**0.5**	**<0.002**	0.06	0.26	<0.01
Oleic acid (18:1 n-9)	**41.1**±**2.8**	**42.3**±**3.0**	**<0.002**	**0.14**	−**0.38**	**<0.002**
Eicosenoic acid (20:1 n-9+11)	**0.5**±**0.1**	**0.6**±**0.1**	**<0.002**	0.00	−0.10	>0.05
						
Polyunsaturated fatty acids	**17.4**±**3.3**	**18.6**±**3.7**	**<0.002**	0.02	−0.15	>0.05
Total n-3 fatty acids	**3.7**±**1.4**	**4.2**±**1.7**	**<0.002**	−0.01	−0.02	>0.05
α-Linolenic acid (18:3 n-3)	1.3±0.4	1.3±0.5	>0.05	0.06	0.27	<0.01
Eicosapentaenoic acid (20:5 n-3)	**0.5**±**0.3**	**0.6**±**0.4**	**<0.002**	−0.01	0.03	>0.05
Docosapentaenoic acid (22:5 n-3)	**0.6**±**0.2**	**0.7**±**0.2**	**<0.002**	**0.10**	−**0.32**	**<0.002**
Docosahexaenoic acid (22:6 n-3)	1.4±0.8	1.6±1.1	<0.05	0.00	−0.08	>0.05
Total n-6 fatty acids	**13.7**±**2.4**	**14.4**±**2.7**	**<0.002**	0.03	−0.20	<0.05
Linoleic acid (18:2 n-6)	**11.9**±**2.3**	**12.4**±**2.6**	**<0.002**	0.03	−0.19	<0.05
γ-linolenic acid (18:3 n-6)	**0.2**±**0.1**	**0.3**±**0.2**	**<0.002**	0.04	0.22	<0.05
Dihomo-γ-linolenic acid (20:3 n-6)	0.3±0.1	0.3±0.1	<0.05	**0.09**	**0.32**	**<0.002**
Arachidonic acid (20:4 n-6)	**1.3**±**0.5**	**1.5**±**0.5**	**<0.002**	0.04	−0.22	<0.05
						
Estimated enzyme activities						
Stearoyl-CoA desaturase 1 (16:1 n-7/16:0)	0.2±0.0	0.2±0.0	<0.05	**0.09**	**0.31**	**<0.002**
Delta-6 desaturase (18:3 n-6/18:2 n-6)	0.0±0.0	0.0±0.0	<0.01	0.03	0.20	<0.05
Delta-5 desaturase (20:4 n-6/20:3 n-6)	5.4±2.2	5.8±1.9	<0.01	**0.16**	−**0.41**	**<0.002**
Elongase (18:0/16:0)	0.1±0.0	0.1±0.0	>0.05	0.03	0.19	<0.05
Elongase (18:1 n-7/16:1 n-7)	**0.6**±**0.1**	**0.7**±**0.1**	**<0.002**	**0.09**	−**0.31**	**<0.002**
DNL	**2.5**±**0.6**	**2.3**±**0.7**	**<0.002**	0.03	0.21	<0.05

Values are presented as mean±s.d.

Statistically significant changes are in bold (*P*<0.002).

**Table 3:** Expression of genes regulating fatty acids metabolism in subcutaneous adipose tissue in the Kuopio Obesity Surgery Study (KOBS, *n*=100)

*Gene*	*Baseline*	*1 year*	P*-value*
*ELOVL1*	**0.0052±0.001**	**0.0045±0.001**	**<0.001**
*ELOVL3*	**0.0002±0.000**	**0.0003±0.000**	**<0.001**
*ELOVL5*	**0.0236±0.009**	**0.0257±0.009**	**<0.05**
*ELOVL6*	**0.0006±0.001**	**0.0012±0.001**	**<0.01**
*ELOVL7*	0.0010**±**0.001	0.0009**±**0.001	>0.05
*FADS1*	0.0019**±**0.001	0.0026**±**0.002	>0.05
*FADS2*	0.0039**±**0.003	0.0042**±**0.003	>0.05
*FADS3*	**0.0219±0.007**	**0.0189±0.007**	**<0.01**
*SCD*	**0.0838±0.065**	**0.1012±0.061**	**<0.001**

Values are presented as a percentage of total transcript reads (mean±s.d.).

Statistically significant changes in bold (*P*<0.05 in paired samples *t*-test).

**Table 4:** Clinical characteristics of the finnish diabetes prevention study (DPS, *n*=122)

	*Baseline*	*1 year*	P*-value*
Sex (men/women)	28/94		
Intervention/Control group	87/35		
Age (years)	54.3±7.1		
Weight (kg)	**88.8±14.7**	**81.4±13.7**	**<0.001**
Body mass index (kg m^−2^)	**32.2±4.6**	**29.5±4.2**	**<0.001**
Fasting Insulin (mU l^−1^)	**14.8±7.7**	**12.6±10.3**	**<0.05**
Fasting glucose (mmol l^−1^)	**6.1±0.5**	**5.8±0.6**	**<0.001**
Total cholesterol (mmol l^−1^)	**5.5±0.8**	**5.4±0.9**	**<0.05**
LDL cholesterol (mmol l^−1^)	3.5±0.7	3.4±0.8	>0.05
HDL cholesterol (mmol l^−1^)	**1.2±0.3**	**1.3±0.3**	**<0.001**
Triglycerides (mmol l^−1^)	**1.7±0.8**	**1.4±0.6**	**<0.001**

Values are presented as mean±s.d.

Statistically significant changes in bold (*P*<0.05 in paired samples *t*-test).
