# Supplementary material for: Alterations in fatty acid metabolism in response to obesity surgery combined with dietary counseling
Source: Nutr Diabetes. 2017 Sep 4;7(9):e285–. doi: 10.1038/nutd.2017.33 (PMC5637104; doi:10.1038/nutd.2017.33)
Supplement: Supplementary Tables [file nutd201733x1.docx]

SUPPLEMENTAL TABLES:

Alterations in fatty acid metabolism in response to obesity surgery combined with dietary counselling.

Paula Walle^1^, Markus Takkunen^1^, Ville Männistö^2^, Maija Vaittinen^1^, Pirjo Käkelä^3^, Jyrki Ågren^4^, Ursula Schwab^1,5^, Jaana Lindström^6^, Jaakko Tuomilehto^6,7,8,9^, Matti Uusitupa^1^, Jussi Pihlajamäki^1,5^

1 Institute of Public Health and Clinical Nutrition, University of Eastern Finland, Finland

2 Department of Medicine, University of Eastern Finland and Kuopio University Hospital, Finland
3 Department of Surgery, University of Eastern Finland and Kuopio University Hospital, Finland

4 Institute of Biomedicine, University of Eastern Finland, Finland

5 Clinical Nutrition and Obesity Center, Kuopio University Hospital, Finland

6 National Institute for Health and Welfare, THL, Finland

7 Center for Vascular Prevention, Danube University Krems, Krems, Austria

8 Diabetes Research Group, King Abdulaziz University, 21589 Jeddah, Saudi Arabia

9 Dasman Diabetes Institute, Dasman, Kuwait
