# Supplementary material for: Alterations in fatty acid metabolism in response to obesity surgery combined with dietary counseling
Source: Nutr Diabetes. 2017 Sep 4;7(9):e285–. doi: 10.1038/nutd.2017.33 (PMC5637104; doi:10.1038/nutd.2017.33)
Supplement: Supplementary Figures [file nutd201733x2.docx]

SUPPLEMENTAL FIGURE:

Alterations in fatty acid metabolism in response to obesity surgery combined with dietary counselling.

Paula Walle^1^, Markus Takkunen^1^, Ville Männistö^2^, Maija Vaittinen^1^, Pirjo Käkelä^3^, Jyrki Ågren^4^, Ursula Schwab^1,5^, Jaana Lindström^6^, Jaakko Tuomilehto^6,7,8,9^, Matti Uusitupa^1^, Jussi Pihlajamäki^1,5^

1 Institute of Public Health and Clinical Nutrition, University of Eastern Finland, Finland

2 Department of Medicine, University of Eastern Finland and Kuopio University Hospital, Finland
3 Department of Surgery, University of Eastern Finland and Kuopio University Hospital, Finland

4 Institute of Biomedicine, University of Eastern Finland, Finland

5 Clinical Nutrition and Obesity Center, Kuopio University Hospital, Finland

6 National Institute for Health and Welfare, THL, Finland

7 Center for Vascular Prevention, Danube University Krems, Krems, Austria

8 Diabetes Research Group, King Abdulaziz University, 21589 Jeddah, Saudi Arabia

9 Dasman Diabetes Institute, Dasman, Kuwait

**Supplemental figure 1.** Correlation between enzyme activities and the amount of weight loss in Kuopio Obesity Surgery study. Panels (A) and (B) for triglycerides, panels (C) and (D) for cholesteryl esters and panel (E) for phospholipids. TG, triglycerides; CE, cholesteryl esters; PL, phospholipids; D5D, delta-5 desaturase; D6D, delta-6 desaturase. Correlation coefficient calculated with Pearson correlation.


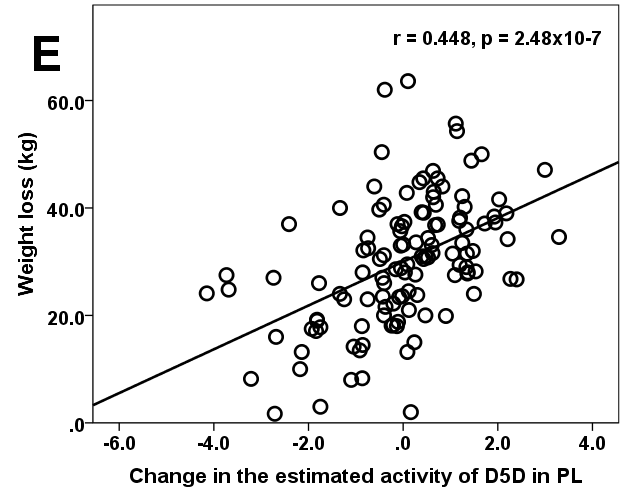

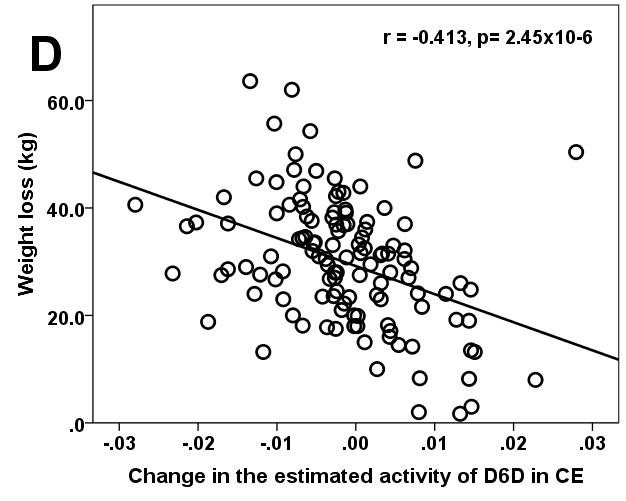

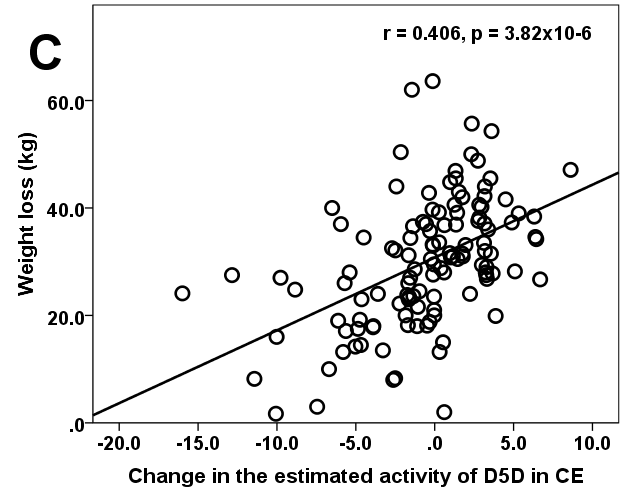

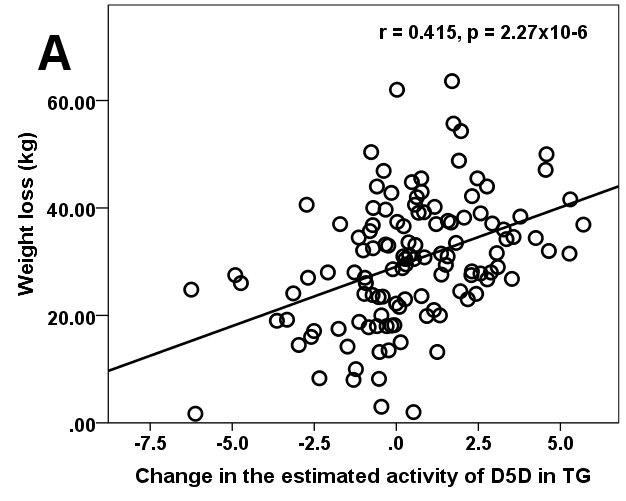

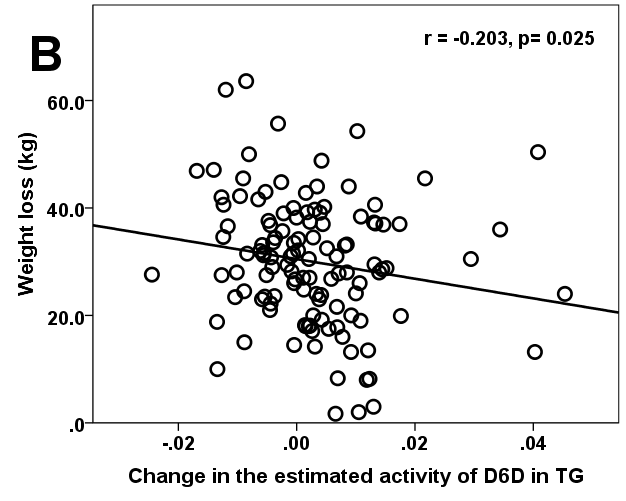


Supplemental figure 1. Correlation between enzyme activities and the amount of weight loss in Kuopio Obesity Surgery study. Panels (A) and (B) for triglycerides, panels (C) and (D) for cholesteryl esters and panel (E) for phospholipids. TG, triglycerides; CE, cholesteryl esters; PL, phospholipids; D5D, delta-5 desaturase; D6D, delta-6 desaturase. Correlation coefficient calculated with Pearson correlation.
